# Iatrogenic propagation of left main coronary artery dissection during diagnostic coronary angiography: A case report

**DOI:** 10.1097/MD.0000000000042040

**Published:** 2025-04-04

**Authors:** Nghia Thuong Nguyen, Nga Anh Thi Nguyen, Hai Phuong Nguyen Tran, Sang Quang Ly

**Affiliations:** a Department of Interventional Cardiology, Cho Ray Hospital, Ho Chi Minh City, Vietnam; b Cardiovascular Research, Methodist Hospital, Merrillville, IN; c Department of Internal Medicine, Faculty of Medicine, University of Medicine and Pharmacy at Ho Chi Minh City, Ho Chi Minh City, Vietnam.

**Keywords:** iatrogenic coronary artery dissection, left main coronary artery dissection

## Abstract

**Rationale:**

Iatrogenic dissection of the left main coronary artery (LMCA) extending to the left anterior descending (LAD) and left circumflex arteries is a very rare but catastrophic complication during coronary intervention. Prompt diagnosis and appropriate management are essential for improving patient outcomes.

**Patient concerns:**

We report the case of a 78-year-old male with a past medical history of hypertension and previous myocardial infarction who presented to the emergency department with progressively worsening angina over 2 weeks.

**Diagnoses:**

During coronary angiography, LMCA dissection occurred due to catheter manipulation. The dissection extended to both the LAD and left circumflex arteries, causing hemodynamic instability.

**Interventions:**

Using a provisional stenting strategy, the dissection was successfully treated with percutaneous transluminal coronary angioplasty and stent placement from the LMCA to the proximal and mid-LAD. Intravascular ultrasound–guided optimization confirmed appropriate stent expansion and apposition after the proximal optimization technique.

**Outcomes:**

The patient’s chest pain resolved postprocedure, and he remained hemodynamically stable during a 6-month follow-up with patent stents confirmed on check angiogram.

**Lessons:**

This case highlights the importance of preventing, recognizing, and promptly managing iatrogenic LMCA dissection to prevent fatal outcomes. Intravascular ultrasound–guided optimization plays a crucial role in ensuring optimal stent placement in these high-risk emergency interventions.

## 1. Introduction

Iatrogenic coronary artery dissection is a rare but serious complication that can occur during percutaneous coronary interventions.^[1]^ Dissection of the left main coronary artery (LMCA) is particularly critical due to its major role in supplying blood to a significant portion of the myocardium. This case report discusses an instance of iatrogenic LMCA dissection extending to the left anterior descending (LAD) and LCx in a 78-year-old male, emphasizing the diagnostic challenges, therapeutic strategies, and clinical implications.

## 2. Case presentation

A 78-year-old male presented to the emergency department with a 1-month history of chest pain that had significantly worsened over the preceding 2 weeks. The chest pain was characterized by tightness in the mid sternum without radiation, triggered by exertion, and relieved by rest, lasting 3 to 5 minutes each episode. The frequency and intensity of these episodes had increased from CCS II to III, prompting his emergency department visit despite the stable angina pattern.

The patient was awake and alert, with mild chest pain. His vital signs included a blood pressure of 110/60 mm Hg, heart rate of 66 bpm, and SpO2 of 96% on room air. His medical history revealed hypertension and an inferior wall myocardial infarction in May 2023, for which he underwent PCI with a DES placement at the mid-RCA. At that time, he was noted to have 80% stenosis in the proximal and mid-LAD, which were left for a staged procedure following a heart team discussion due to the complexity of the coronary anatomy and the patient’s clinical stability.

He was on medications including Clopidogrel/Aspirin 75/100 mg once daily, Rosuvastatin 20 mg once daily, Bisoprolol 2.5 mg once daily, Esomeprazole 40 mg once daily, Candesartan 8 mg once daily, and Isosorbide 5-mononitrate 60 mg once daily.

### 2.1. Laboratory and diagnostic findings

Laboratory tests revealed hemoglobin at 126 g/L, white blood cell count of 12.5 g/L, neutrophils at 67.6%, platelets at 282 g/L, INR of 1.04, PT of 11.4 seconds, aPTT of 31.9 seconds, fibrinogen at 3.5 g/L, glucose at 178 mg/dL, AST at 19 U/L, ALT at 32 U/L, creatinine at 1.01 mg/dL, and an estimated glomerular filtration rate (eGFR) of 81.61 mL/min/1.73 m^2^. Electrolytes showed sodium at 136 mmol/L, potassium at 3.6 mmol/L, and chloride at 101 mmol/L. Lipid profile results included total cholesterol at 144 mg/dL, triglycerides at 162 mg/dL, LDL-c at 88 mg/dL, and HDL-c at 34 mg/dL. Cardiac enzymes were CK-MB at 20.69 U/L and high-sensitivity cardiac troponin I at 34.83 pg/mL.

**Figure 1.**
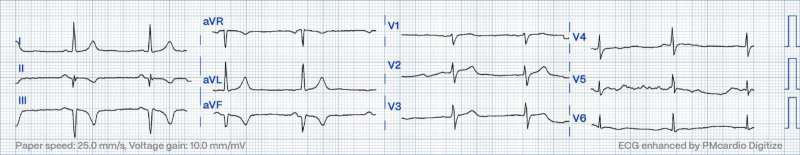
The patient’s ECG showed sinus bradycardia, negative T waves in leads DII, III, and aVF. aVF = augmented vector foot, ECG = electrocardiography.

Electrocardiography (ECG) showed sinus bradycardia and negative T in leads DII, III, and augmented vector foot (Fig. 1). Echocardiography showed no regional wall motion abnormalities and preserved left ventricular systolic function with an ejection fraction of 51%.

The patient was diagnosed with a subacute inferior wall myocardial infarction with a DES placement in the mid-RCA and significant stenosis (80%) in the proximal and mid-LAD—hypertension. Revascularization with PCI of the remaining lesion in the LAD is planned to be performed.

### 2.2. Procedural details and complication management

The procedure began with imaging of RCA using a standard JR 4.0 5F catheter, which revealed that the previously placed DES in the mid-RCA was patent, with 50% stenosis in the distal RCA and no significant restenosis or new lesions. Next, the LCA intervention was performed using an EBU 3.5 6F catheter.

**Figure 2.**
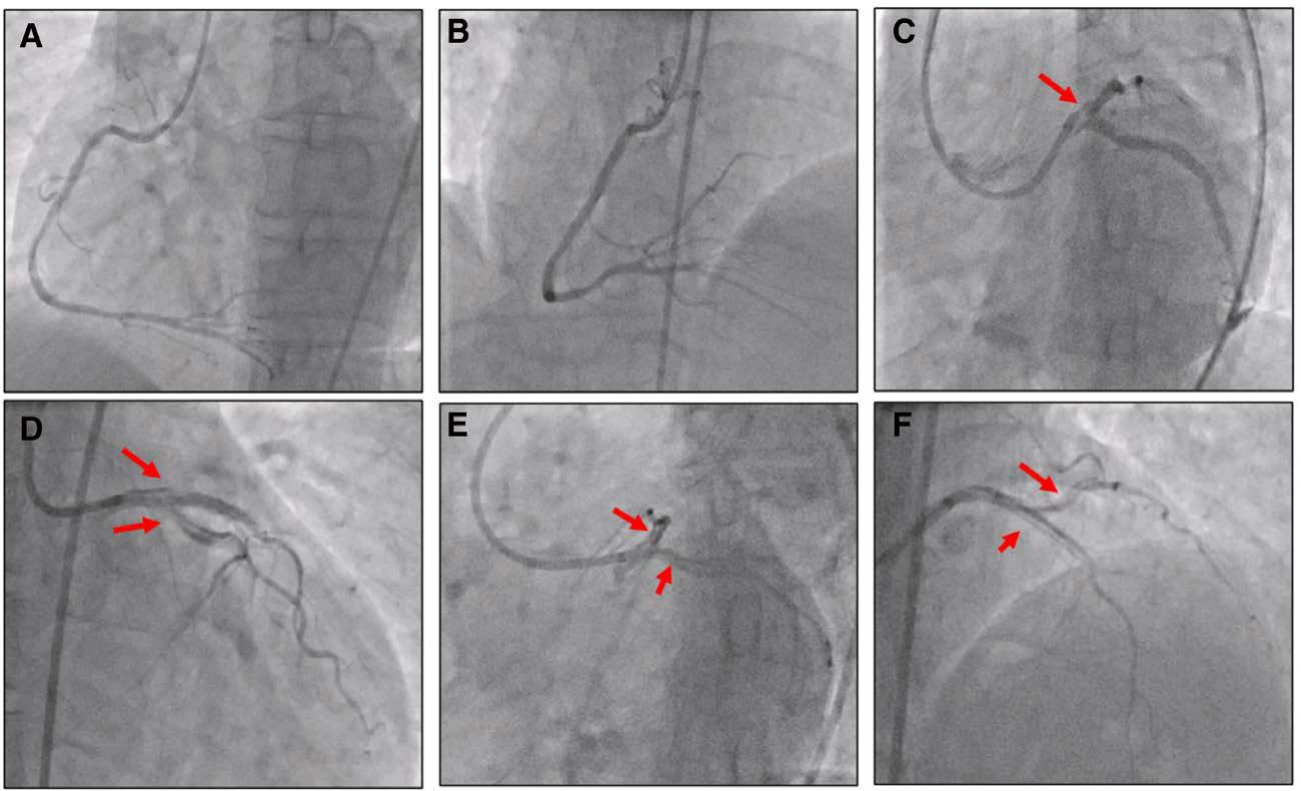
Coronary angiography images of the patient. (A, B) The previously placed stent in the mid-RCA was patent, with 50% stenosis noted in the distal RCA. (C) The EBU catheter was not aligned coaxially with the LMCA, resulting in a dissection of the LMCA when contrast was injected (red arrow). (D, E, and F) The dissection extended longitudinally into both the LAD and LCx (red arrows), compromising blood flow with a TIMI flow grade of II and III. LAD = left arterial descending, LMCA = left main coronary artery, RCA = right coronary artery, TIMI = Thrombolysis in Myocardial Infarction.

During the procedure, while installing the EBU catheter into the left main artery and performing a contrast injection for imaging, a dissection of the LM occurred, and the patient complained of chest pain again, with blood pressure dropping to 70 mm Hg, and ST elevation changes on the monitor. The dissection spread vertically to both LAD and LCx, resulting in Thrombolysis in Myocardial Infarction (TIMI) flow grades II and III. Additionally, there was 80% stenosis in the mid-LAD (Fig. 2, Video S1, Supplemental Digital Content, http://links.lww.com/MD/O609).

We attempted to navigate the true lumen using a Runthrough floppy wire 0.014’’. Although a microcatheter could have provided additional support for wire manipulation and confirmation of true lumen positioning, the emergency nature of the situation prompted direct wire navigation. With careful manipulation, we successfully positioned the wire into the true lumen of the LMCA to distal LAD. We secured a second Runthrough floppy wire in the true lumen of the LCx using the same technique.

**Figure 3.**
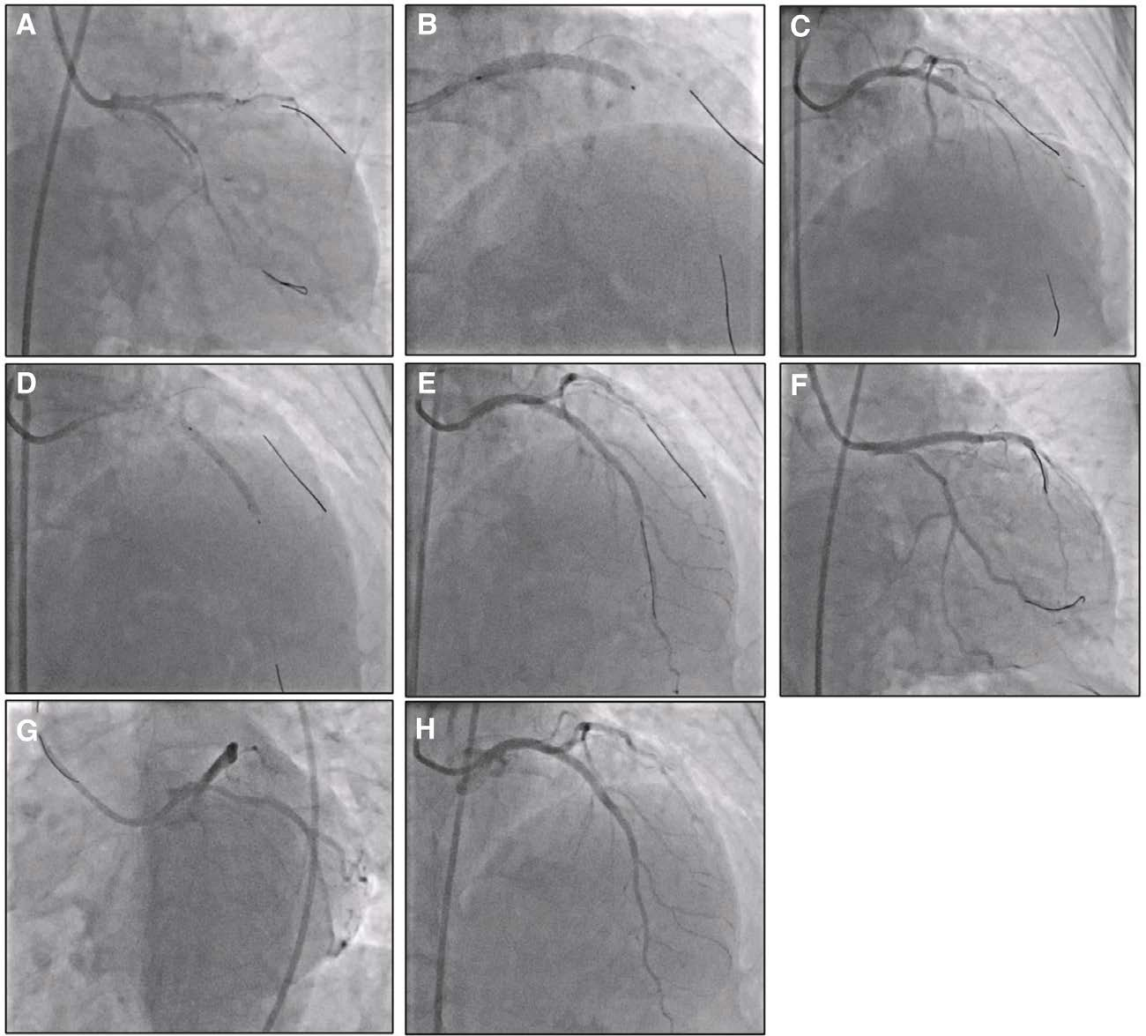
PTCA was performed on this patient. PTCA = percutaneous transluminal coronary angioplasty. (A) 2 wires were secured in the true lumen of both LAD and LCx. (B) LMCA-proximal LAD stenting with an Ultimaster Tansei 3.5 × 33 mm. (C) After stenting, TIMI flow grade II was observed with severe mid-LAD stenosis. (D) Mid-LAD stenting with a Biomime Morph 3.0 × 30 mm. (E, F, G, and H) The LM-LAD-LCx dissection was contained, and blood flow improved with a TIMI grade III. LAD = left arterial descending, LMCA = left main coronary artery, TIMI = Thrombolysis in Myocardial Infarction.

After confirming wire positions in both the LAD and LCx, we employed a provisional stenting strategy (crossover technique) due to the emergency nature of the situation and the relatively minimal involvement of the LCx with preserved TIMI III flow. Rapid stenting of the LMCA to the proximal LAD was performed using an Ultimaster Tansei 3.5 × 33 mm stent deployed at 12 atm, achieving a final diameter of 3.62 mm, with a wire jailed in the LCx. This was followed by proximal optimization technique with a Wilma NC 4.0 × 12 mm balloon inflated to 14 atm. Additionally, PTCA was performed on the mid-LAD with a Biomine Morph 3.0 × 30 mm stent deployed at 12 atm, achieving a final diameter of 3.14 mm. The condition of LM-LAD-LCx dissection was immediately contained and improved, as observed through angiography with TIMI flow grade III (Fig. 3, Video S2, Supplemental Digital Content, http://links.lww.com/MD/O610 and Video S3, Supplemental Digital Content, http://links.lww.com/MD/O611).

**Figure 4.**
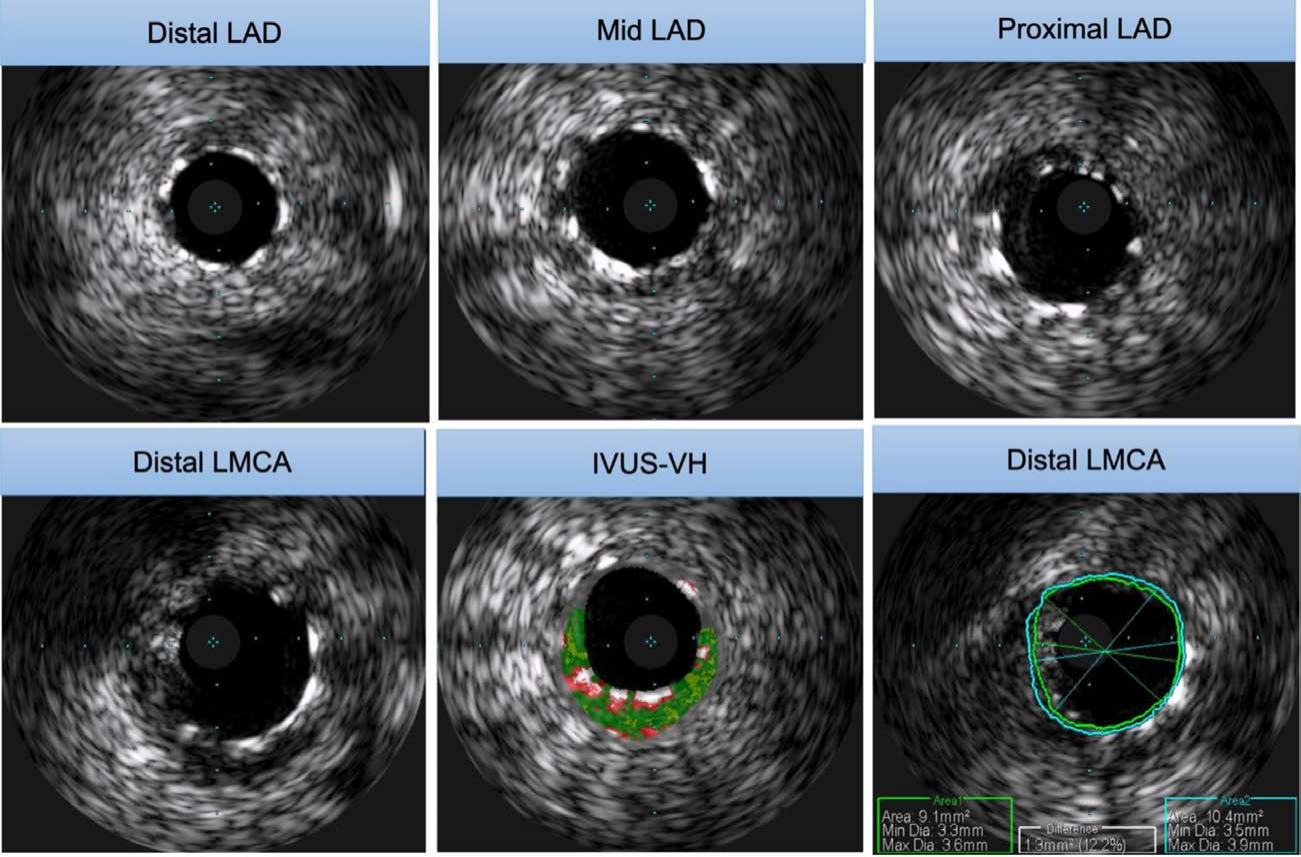
IVUS confirmed that the stents were well opposed to the vessel walls, and the dissection was contained without further propagation.

Following the intervention, IVUS was utilized to assess the positioning of the stents and the extent of the dissection. IVUS confirmed that the stents were well apposed to the vessel walls, and the dissection was contained without any further propagation. This imaging modality provided detailed visualization, ensuring the adequacy of the stent deployment and the stability of the arterial wall post-procedure (Fig. 4, Video S4, Supplemental Digital Content, http://links.lww.com/MD/O612).

The patient was already on Clopidogrel/Aspirin 75/100mg daily as part of his post-MI regimen from May 2023. During the procedure, an intravenous bolus of unfractionated heparin (100 IU/kg) was administered.

### 2.3. Post-procedural course and follow-up

The patient’s immediate outcome was favorable, with resolved chest pain and stable hemodynamics. Long-term management included continued dual antiplatelet therapy (Clopidogrel/Aspirin 75/100 mg daily), statin (Rosuvastatin 20 mg daily), beta-blocker (Bisoprolol 2.5 mg daily), and other supportive medications to ensure optimal cardiac function and prevent further ischemic events.

The patient was followed for 6 months after the procedure with clinical evaluations at 1, 3, and 6 months, including ECG and echocardiography at each visit. A repeat angiogram performed at 6 months post-procedure showed patent stents in the LMCA, LAD, and RCA with no evidence of restenosis, complete healing of the previously dissected segments, no new lesions, and preserved TIMI III flow in all vessels. The patient remained asymptomatic throughout the follow-up period with good exercise tolerance and no recurrence of angina.

## 3. Discussion

Iatrogenic dissection of the LMCA is a rare but potentially catastrophic complication that can occur during PCI. This condition is particularly dangerous due to the critical role of the LMCA in supplying blood to a large portion of the myocardium. Dissections in this artery can quickly lead to significant myocardial ischemia, ventricular arrhythmias, cardiogenic shock, or even sudden cardiac death if not promptly recognized and managed. The incidence of iatrogenic LMCA dissection ranges from 0.07% to 0.2% during coronary angiography and PCI, emphasizing the need for high vigilance and immediate intervention strategies.^[1]^

### 3.1. Mechanism

In this case, the dissection occurred during the noncoaxial insertion of an EBU 3.5 6F catheter into the LMCA and the subsequent contrast injection for imaging. The mechanical force exerted by the catheter on the arterial wall, combined with high-pressure contrast injection, likely resulted in a tear in the intimal layer of the LMCA. This tear propagated vertically, extending into both the LAD and left circumflex arteries. The resultant disruption in the integrity of the vessel wall compromised coronary blood flow, indicated by the TIMI flow grades II and III observed in the angiographic images.

### 3.2. Diagnostic approach

The rapid identification of the dissection was crucial in this patient’s management. The rapid identification of the dissection was crucial in this patient’s management. In this case, the patient developed sudden-onset chest pain, hypotension (blood pressure dropping to 70 mm Hg), and ST-segment elevation on continuous ECG monitoring, suggesting a newly occurring acute event that requires urgent diagnosis.

Angiographically, dissection appears as a radiolucent line within the contrast-filled lumen, haziness, or abrupt vessel cutoff. Our patient presented with a type C dissection, characterized by persistent extraluminal contrast staining.^[2]^

### 3.3. Management strategy

The primary therapeutic goal in managing iatrogenic LMCA dissection is to restore coronary flow and seal the dissection entry point. The strategy includes securing the wire in the true lumen, which is the most important step in managing a dissection, followed by stent deployment to cover the dissected segments.^[2,3]^

In our case, we employed a provisional stenting strategy (crossover technique) with placement of the first stent from the LMCA to proximal LAD with a wire jailed in the LCx. This approach was chosen due to the emergency nature of the situation, the minimal involvement of the LCx with preserved TIMI III flow, and the patient’s hemodynamic instability necessitating rapid resolution.^[2]^

Wire manipulation is critical in these scenarios. The use of a soft, hydrophilic guidewire (Runthrough floppy wire in our case) facilitated navigation into the true lumen. While we did not use a microcatheter in this emergency situation, such devices can provide additional support for wire manipulation and confirmation of true lumen positioning through contrast injection. This technical consideration should be kept in mind for similar cases when time permits.

Post-intervention, IVUS was utilized to evaluate the positioning of the stents and the extent of the dissection. IVUS provided high-resolution images of the vessel wall, confirming that the stents were well apposed and the dissection was successfully contained. This imaging modality is invaluable in confirming the success of the intervention, as it can detect issues such as malposition or incomplete stent expansion that may not be visible on angiography alone.^[2,4]^

### 3.4. Long-term outcomes and follow-up

Long-term outcomes after iatrogenic LMCA dissection depend on the extent of myocardial damage and the success of the initial intervention. Reported in-hospital mortality rates range from 3% to 12%, highlighting the critical nature of this complication.^[5]^ Successful stenting with restoration of TIMI III flow, as achieved in our case, is associated with improved survival rates.

Regular follow-up with clinical assessment, noninvasive testing, and angiographic evaluation is essential to monitor for stent patency and detect potential complications. Our patient underwent follow-up angiography at 6 months, which confirmed stent patency and complete healing of the dissected segments, with no evidence of restenosis or new lesions.

### 3.5. Lessons

This case highlights several critical lessons that can inform future practice. First and foremost, early recognition of iatrogenic dissection is paramount. Operators must maintain high vigilance during coronary interventions, particularly when manipulating catheters and injecting contrast media, as these actions can precipitate dissections. The prompt identification of dissection, as demonstrated in this case, enables timely intervention, which is crucial for preserving myocardial function and preventing catastrophic outcomes.^[5]^ Additionally, the use of advanced imaging techniques such as IVUS plays a pivotal role in diagnosing and managing coronary dissections. IVUS provides high-resolution images that confirm stent apposition and detect any residual dissection or malposition that might not be visible on standard angiography. This ensures the adequacy of the intervention and guides further therapeutic decisions. Moreover, a well-planned and executed intervention strategy is essential. This includes the careful selection and deployment of stents, as well as post-dilation, to secure the dissected segments and restore optimal blood flow. Finally, continuous patient monitoring and regular follow-up are critical to assess stent patency, detect potential complications, and ensure long-term success. This comprehensive approach underscores the importance of multidisciplinary teamwork and the integration of advanced diagnostic tools in managing complex coronary interventions.^[2,6]^

## 4. Conclusion

This case report underscores the critical nature of iatrogenic propagation LMCA dissection and the importance of prevention, early intervention, and advanced imaging techniques in managing such cases. Recognition of this condition and prompt and appropriate interventional strategies are vital to improve patient outcomes. The use of IVUS, in this case, highlights its value in confirming the adequacy of the intervention and ensuring patient safety.

## Acknowledgments

We greatly appreciate the participation of the relevant clinicians, as well as the imaging and laboratory technicians.

## Author contributions

**Formal analysis:** Nghia Thuong Nguyen, Sang Quang Ly.

**Writing – original draft:** Nghia Thuong Nguyen, Sang Quang Ly, Nga Anh Thi Nguyen, Hai Phuong Nguyen Tran.

**Writing – review & editing:** Nghia Thuong Nguyen, Sang Quang Ly, Nga Anh Thi Nguyen, Hai Phuong Nguyen Tran.

## Supplementary Material


